# Development and Physicochemical Characterization of Trans-Resveratrol Liposomes: Influence of Phospholipid Composition and Ultrasound-Assisted Processing

**DOI:** 10.3390/pharmaceutics18101303

**Published:** 2026-10-14

**Authors:** Lauryna Pudžiuvelytė, Agnė Paliulian

**Affiliations:** 1 Institute of Pharmaceutical Technologies, Lithuanian University of Health Sciences, LT-50161 Kaunas, Lithuania; agne.paliulian@stud.lsmu.lt; 2 Department of Drug Technology and Social Pharmacy, Lithuanian University of Health Sciences, LT-50161 Kaunas, Lithuania

**Keywords:** trans-resveratrol, liposomes, phospholipids, ethanol injection, ultrasound-assisted processing, ultrasonic bath, particle size, polydispersity index, zeta potential, stability

## Abstract

**Background/Objectives:** Trans-resveratrol is a bioactive polyphenol with limited pharmaceutical applicability due to poor aqueous solubility, chemical instability, and low bioavailability. This study aimed to develop trans-resveratrol-containing liposomal formulations and evaluate the influence of phospholipid composition and ultrasound-assisted processing (UAP) using a conventional ultrasonic bath on their physicochemical properties and storage behavior. **Methods:** Liposomes were prepared by the ethanol injection method using Lipoid^®^ S75, Lipoid^®^ P45, soybean lecithin, egg yolk lecithin, and selected binary phospholipid mixtures. Trans-resveratrol identity was verified by LC–MS. Particle size, polydispersity index (PDI), and zeta potential were determined before and after UAP, and physical stability was assessed after 30 days of refrigerated, light-protected storage. **Results:** Phospholipid composition markedly influenced liposome physicochemical characteristics. Lipoid^®^ P45-based formulations exhibited favorable initial particle size, PDI, and zeta potential profiles. UAP generally reduced particle size and improved particle size distribution immediately after preparation, although the magnitude of the effect depended on formulation composition. During refrigerated storage, short-term physical storage behavior was strongly formulation-dependent. Selected trans-resveratrol-containing formulations, particularly Lr12, Lr13, and Lr15, exhibited marked particle growth and increased polydispersity, whereas Lr20–Lr22 exhibited the most favorable particle size and PDI profiles after 30 days. **Conclusions:** Phospholipid composition was a critical determinant of trans-resveratrol liposome performance. Conventional ultrasonic-bath processing provided a simple and accessible means of improving initial liposomal characteristics, but did not universally ensure short-term physical stability during 30 days of refrigerated storage. Selected binary phospholipid formulations, particularly Lr20–Lr22, showed favorable physicochemical profiles and may represent promising candidates for further formulation development.

## 1. Introduction

Liposomes are among the most extensively investigated nanocarrier systems for the delivery of bioactive compounds with limited aqueous solubility and poor physicochemical stability. Their phospholipid bilayer structure enables the encapsulation of both hydrophilic and lipophilic molecules, providing protection against environmental degradation, improving aqueous dispersion, and potentially enhancing bioavailability [1,2]. Owing to their excellent biocompatibility, biodegradability, and versatility, liposomes have been widely explored for pharmaceutical, nutraceutical, and cosmetic applications [3,4,5].

Trans-resveratrol (3,5,4′-trihydroxystilbene) is a naturally occurring polyphenolic compound predominantly found in grapes, berries, peanuts, and red wine [6,7]. It has attracted considerable attention because of its antioxidant, anti-inflammatory, cardioprotective, neuroprotective, antimicrobial, and anticancer activities [8,9,10,11,12]. Despite these promising pharmacological properties, the clinical and pharmaceutical application of trans-resveratrol remains limited by its poor aqueous solubility, susceptibility to photoisomerization and oxidation, rapid metabolism, and consequently low oral bioavailability [12,13]. These limitations substantially reduce its therapeutic potential and have stimulated intensive research into alternative delivery systems capable of improving its stability and bioavailability [14].

Among the various nanocarrier platforms investigated for trans-resveratrol delivery, liposomes represent one of the most promising approaches because the lipophilic structure of trans-resveratrol allows its incorporation into the phospholipid bilayer, thereby protecting the compound from degradation while improving its dispersion in aqueous media. Previous studies have demonstrated that liposomal encapsulation may enhance the physicochemical stability, controlled release, and biological activity of trans-resveratrol [15,16,17,18]. However, the characteristics of liposomal formulations are highly dependent on formulation variables, including phospholipid composition, preparation method, and post-processing conditions.

Phospholipid composition plays a fundamental role in liposome formation and stability. Differences in phosphatidylcholine content, fatty acid composition, and lipid purity influence membrane fluidity, vesicle formation, particle size, surface charge, and long-term stability [19]. Commercial phospholipids such as Lipoid^®^ S75, Lipoid^®^ P45, soybean lecithin, and egg yolk lecithin differ considerably in their composition and may therefore produce liposomes with distinct physicochemical characteristics. Although these phospholipids have been used individually in liposomal formulations, comparative studies evaluating their performance under identical formulation conditions remain limited.

Besides lipid composition, post-formulation processing is another important factor affecting liposome quality. Various size-reduction techniques, including extrusion, high-pressure homogenization, and probe ultrasound homogenization, have been employed to obtain nanosized liposomes with narrow particle size distributions [20]. However, these techniques frequently require specialized equipment and relatively high operating costs. In contrast, ultrasound-assisted processing using a conventional ultrasonic bath represents a simple, inexpensive, and widely accessible alternative that may improve liposomal dispersions without the need for sophisticated instrumentation. Despite its routine use in laboratory practice, its influence on the physicochemical characteristics of trans-resveratrol liposomes has received comparatively little attention.

Particle size, PDI, and zeta potential are considered critical quality attributes of liposomal formulations because they directly influence colloidal stability, reproducibility, storage behavior, and potential biological performance [21,22]. Therefore, systematic evaluation of these parameters is essential for selecting suitable phospholipid systems and assessing the effectiveness of post-formulation processing.

The present study aimed to develop trans-resveratrol-containing liposomal formulations using different natural phospholipid compositions prepared by the ethanol injection method and to evaluate the influence of ultrasound-assisted processing using a conventional ultrasonic bath on their physicochemical characteristics and short-term physical storage behavior. In addition, control liposomal formulations without trans-resveratrol were included to distinguish the effects of phospholipid composition from those associated with the presence of trans-resveratrol in the formulation. The findings provide practical guidance for the development of simple and reproducible liposomal formulations using an accessible post-processing approach.

## 2. Materials and Methods

### 2.1. Materials

Trans-resveratrol (≥98% purity) was used as the model lipophilic bioactive compound in all formulations. Prior to liposome preparation, its identity was verified by liquid chromatography–mass spectrometry (LC–MS).

Four natural phospholipid materials with different phosphatidylcholine contents were selected for liposome preparation to evaluate the influence of lipid composition on the physicochemical characteristics of the formulations. Lipoid^®^ S75 (≥70% phosphatidylcholine) and Lipoid^®^ P45 (≥45% phosphatidylcholine) were obtained from Lipoid GmbH (Ludwigshafen, Germany). Soybean lecithin and egg yolk lecithin were purchased from Sigma-Aldrich (St. Louis, MO, USA). Ethanol (96%, pharmaceutical grade) was obtained from Stumbras (Kaunas, Lithuania), while purified water was produced using a Milli-Q water purification system (Millipore, Bedford, MA, USA).

The physicochemical properties of the liposomal formulations were determined using a Zetasizer Nano ZS (Malvern Instruments Ltd., Malvern, UK), which employs dynamic light scattering (DLS) for particle size and PDI measurements and electrophoretic light scattering (ELS) for zeta potential determination. Ultrasound bath-assisted processing was performed using a SONOREX DIGITEC DT 156 BH ultrasonic bath (BANDELIN electronic GmbH & Co. KG, Berlin, Germany), and lipid dispersions were prepared using an IKA C-MAG HS7 magnetic stirrer (IKA-Werke GmbH & Co. KG, Staufen, Germany) and a Heidolph Laborota 4000 efficient rotary evaporator (Heidolph Instruments GmbH & Co. KG, Schwabach, Germany). Lipid solutions were introduced into the aqueous phase using a Landgraf LA-120 syringe pump (Landgraf Laborsysteme HLL GmbH, Langenhagen, Germany). Sample preparation was performed using calibrated analytical balances (Shimadzu AUW120D, Kyoto, Japan) and adjustable micropipettes (Eppendorf AG, Hamburg, Germany).

All chemicals and reagents were of analytical or pharmaceutical grade and were used as received without further purification.

### 2.2. Characterization of Trans-Resveratrol

The identity of trans-resveratrol was qualitatively verified prior to formulation by UPLC–MS/MS. Chromatographic separation was performed using electrospray ionization in the negative ion mode (ESI−). Qualitative identification was based on the chromatographic retention behavior together with the diagnostic MRM transition *m*/*z* 227 → 185 under the analytical conditions described in Section 2.6. The representative chromatogram exhibited a predominant peak at a retention time of approximately 6.67 min, and no major interfering peaks were observed.

The chromatographic analysis was used for qualitative identity verification of the trans-resveratrol starting material before liposome preparation and was not used to quantify trans-resveratrol in the liposomal formulations.

### 2.3. Preparation of Liposomal Formulations

Trans-resveratrol-containing liposomal formulations were prepared using the ethanol injection method. Four phospholipid materials were investigated: Lipoid S75 and Lipoid P45 (Lipoid GmbH, Germany), L-α-phosphatidylcholine from soybean, Type IV-S (P3644, Sigma-Aldrich), and L-α-phosphatidylcholine from egg yolk (61755, Sigma-Aldrich). Their compositional characteristics, including phosphatidylcholine content, reported fatty acid composition, degree of saturation, and available manufacturer specifications, are summarized in Table 1.

Phospholipids were dissolved in 20 mL of 96% (*v*/*v*) ethanol under continuous magnetic stirring (700 rpm) at 37 ± 1 °C until a clear and homogeneous lipid solution was obtained. Trans-resveratrol was subsequently dissolved in the lipid phase according to the composition of each formulation.

The ethanolic lipid solution was slowly injected into the aqueous phase (20 mL) using a syringe pump at a constant flow rate of 300 mL h^−1^ while the aqueous phase was continuously stirred at 200 rpm. The rapid diffusion of ethanol into the aqueous medium induced spontaneous self-assembly of phospholipids, resulting in the formation of liposomal dispersions. After complete addition of the lipid phase, the dispersions were stirred for an additional 2–3 min to ensure complete vesicle formation.

The dispersion (40 mL) was concentrated under reduced pressure at 40 °C to a final volume of 20 mL using a Heidolph Laborota 4000 efficient rotary evaporator (Heidolph Instruments GmbH, Schwabach, Germany) at 250 rpm under reduced pressure to remove the majority of ethanol from the formulation. The resulting 20 mL liposomal dispersion was used for subsequent physicochemical characterization and further experimental procedures. The residual ethanol concentration in the final formulation was not quantitatively determined.

To evaluate the influence of a simple post-formulation processing step on liposomal quality, each freshly prepared formulation was divided into two equal 10 mL aliquots. One aliquot was characterized immediately after preparation, whereas the second aliquot was subjected to ultrasound-assisted processing using a SONOREX DIGITEC DT 156 BH ultrasonic bath (BANDELIN electronic GmbH & Co. KG, Berlin, Germany) operating at an ultrasonic frequency of 35 kHz, with a nominal ultrasonic power of 215 W and a peak ultrasonic power of 860 W. The bath was filled with 6 L of purified water. For each treatment, 10 mL of liposomal dispersion was transferred into a screw-capped glass vial. The sealed vials were placed horizontally in the ultrasonic bath and sonicated continuously for 30 min. The bath temperature was set to 25 °C. An additional electronic thermometer was placed in the bath to independently monitor the water temperature during treatment and to ensure that no substantial temperature increase occurred during sonication.

This approach was selected as a simple, low-cost, and readily accessible alternative to high-energy size-reduction techniques, such as probe sonication or high-pressure homogenization.

Following preparation, liposomal dispersions were transferred into amber glass containers to minimize light-induced degradation of trans-resveratrol. Samples intended for stability studies were stored at 5 ± 3 °C until further physicochemical characterization.

### 2.4. Formulation Composition

A total of 22 liposomal formulations were prepared. Eight control formulations (C1–C8) contained phospholipids without trans-resveratrol, whereas fourteen formulations (Lr9–Lr22) were prepared with nominal trans-resveratrol amounts of 100 or 200 mg. These values represent the nominal amounts of trans-resveratrol added during formulation and should not be interpreted as the amount encapsulated or incorporated into the liposomal system. Four phospholipid materials and selected binary phospholipid mixtures were investigated (Table 2).

### 2.5. Physicochemical Characterization

#### 2.5.1. Mean Particle Size and Polydispersity Index

The mean particle size and PDI of liposomal dispersions were determined by dynamic light scattering (DLS) using a Zetasizer Nano ZS (Malvern Instruments Ltd., Malvern, UK).

Prior to DLS measurements, the liposomal dispersions were diluted with 5% (*v*/*v*) aqueous ethanol at a ratio of 50 μL sample to 950 μL diluent to obtain an appropriate particle concentration for reliable analysis and to minimize concentration-dependent multiple-scattering effects. The same dilution procedure and dilution medium were used for all formulations. Measurements were performed in disposable polystyrene cuvettes following equilibration at 25 °C. Scattered light intensity was recorded at a detection angle of 173°. Each formulation was analyzed before and after UAP, and all measurements were performed in four replicates (*n* = 4).

#### 2.5.2. Zeta Potential

The surface charge of liposomal particles was evaluated by electrophoretic light scattering (ELS) using the M3-PALS technique implemented in the Zetasizer Nano ZS. Samples were diluted under the same conditions as described for particle size measurements and transferred into folded capillary cells. Following equilibration at 25 °C, zeta potential measurements were performed at a scattering angle of 90°. Measurements were conducted before and after UAP to determine the influence of post-processing on colloidal stability.

### 2.6. Qualitative UPLC–MS/MS Characterization of Trans-Resveratrol

Qualitative characterization of trans-resveratrol was performed using an Acquity H-Class ultra-performance liquid chromatography (UPLC) system coupled to a Xevo TQD tandem mass spectrometer (Waters, Milford, MA, USA). Chromatographic separation was carried out using a YMC-Triart C18 column (100 × 2.0 mm, 1.9 µm, 100 Å; YMC, Kyoto, Japan) equipped with a guard column and maintained at 40 °C.

The mobile phase consisted of solvent A, 0.1% (*v*/*v*) aqueous formic acid, and solvent B, acetonitrile. The initial conditions of 95% A and 5% B were maintained for 1 min. The proportion of solvent A was then decreased to 70% by 5 min and to 50% by 7 min. From 7.5 to 8.0 min, the mobile phase consisted of 100% B, after which the system was returned to the initial conditions at 8.1 min. The total chromatographic run time was 10 min. The flow rate was 0.5 mL min^−1^, and the injection volume was 1 µL.

Mass-spectrometric detection was performed in negative electrospray ionization mode. The capillary voltage was set to 2.5 kV, the source temperature to 120 °C, and the desolvation temperature to 500 °C. The desolvation gas flow rate was 800 L h^−1^. Trans-resveratrol was monitored in multiple reaction monitoring (MRM) mode using the transition *m*/*z* 227 → 185. The UPLC–MS/MS procedure was used for qualitative identity confirmation only; quantitative determination of trans-resveratrol in the liposomal formulations was not performed in this analysis.

### 2.7. Assessment of Short-Term Physical Storage Stability

The short-term physical stability of both control and trans-resveratrol-containing liposomal formulations was evaluated during 30 days of refrigerated storage.

Freshly prepared dispersions were transferred into sealed amber glass vials and stored at 5 ± 3 °C, protected from light. Physicochemical characterization, including particle size, PDI, and zeta potential, was repeated after 30 days of storage. The obtained values were compared with the initial measurements performed immediately after formulation preparation to assess changes in physicochemical characteristics and short-term colloidal storage behavior over time.

### 2.8. Statistical Analysis

All experimental data are presented as mean ± standard deviation (SD) from four independent formulation replicates (*n* = 4). Statistical comparisons were performed within each formulation to evaluate the effects of UAP and refrigerated storage. The effect of UAP was assessed by comparing paired measurements obtained before and after UAP within the same formulation and at the same storage time point. The effect of storage was assessed by comparing paired measurements obtained at day 0 and day 30 within the same formulation and under the same UAP condition. Paired Student’s *t*-tests were used for within-formulation comparisons. To account for multiple testing across formulations, *p*-values were adjusted using the Holm method within each family of comparisons. Holm-adjusted *p*-values < 0.05 were considered statistically significant. Comparisons between different formulations were not included in the inferential statistical analysis, as the primary objective was to evaluate UAP- and storage-induced changes within individual formulations rather than to statistically rank formulations. Statistical analyses and graphical presentation of the data were performed using Microsoft Excel 2016 (Microsoft Corp., Redmond, WA, USA).

## 3. Results

### 3.1. Qualitative Identification of Trans-Resveratrol by UPLC–MS/MS

**Figure 1:**
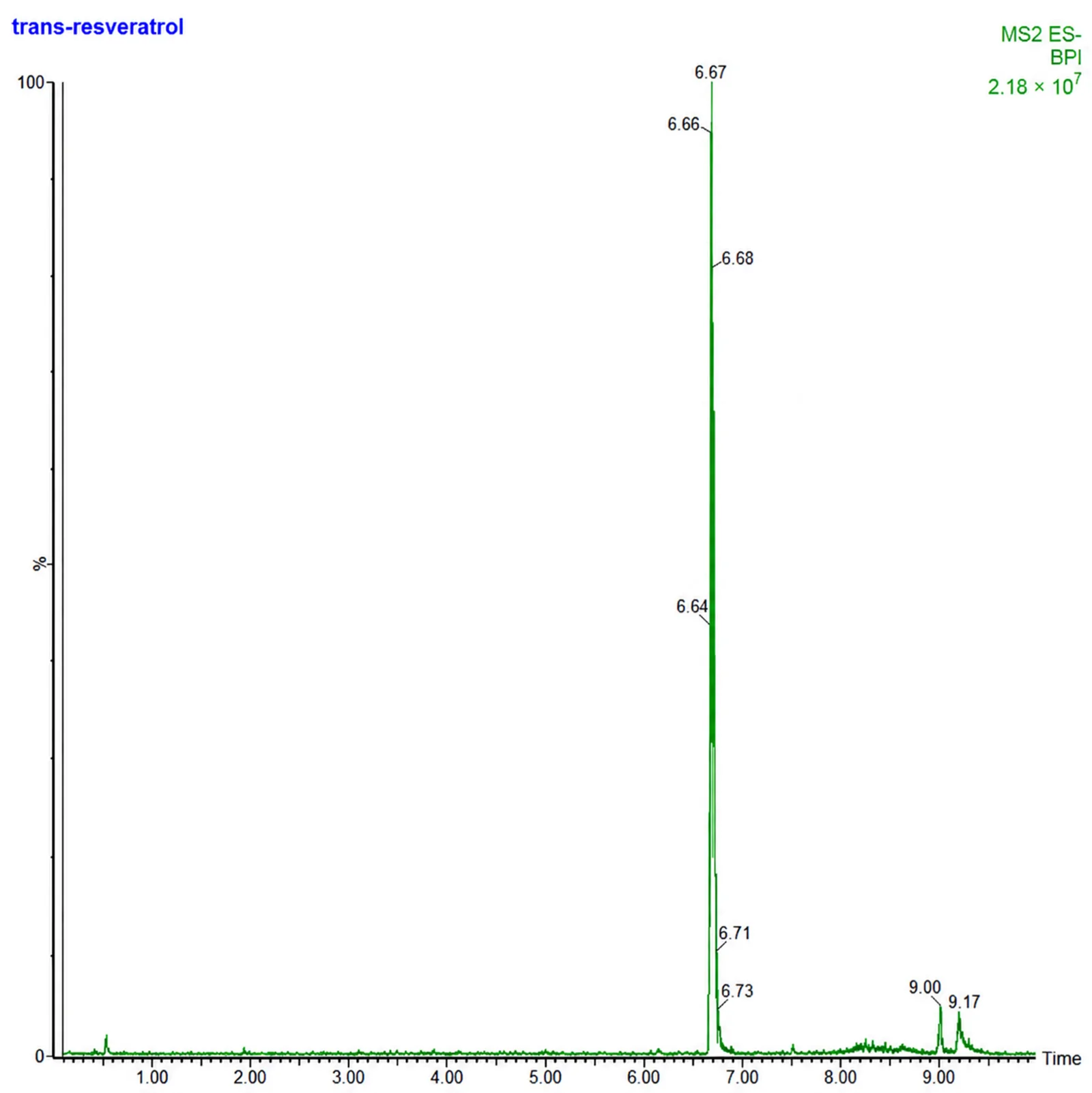
Representative UPLC–MS/MS chromatogram of trans-resveratrol.

The identity of trans-resveratrol used for liposome preparation was qualitatively verified by UPLC–MS/MS prior to formulation. The representative chromatogram (Figure 1) exhibited a dominant peak at a retention time of approximately 6.67 min, together with the diagnostic MRM transition *m*/*z* 227 → 185.

No additional major peaks were evident under the applied analytical conditions. The UPLC–MS/MS profile was consistent with the expected identity of trans-resveratrol and supported its use in subsequent formulation studies.

### 3.2. Effect of Phospholipid Composition on the Physicochemical Properties of Control Liposomes

**Figure 2:**
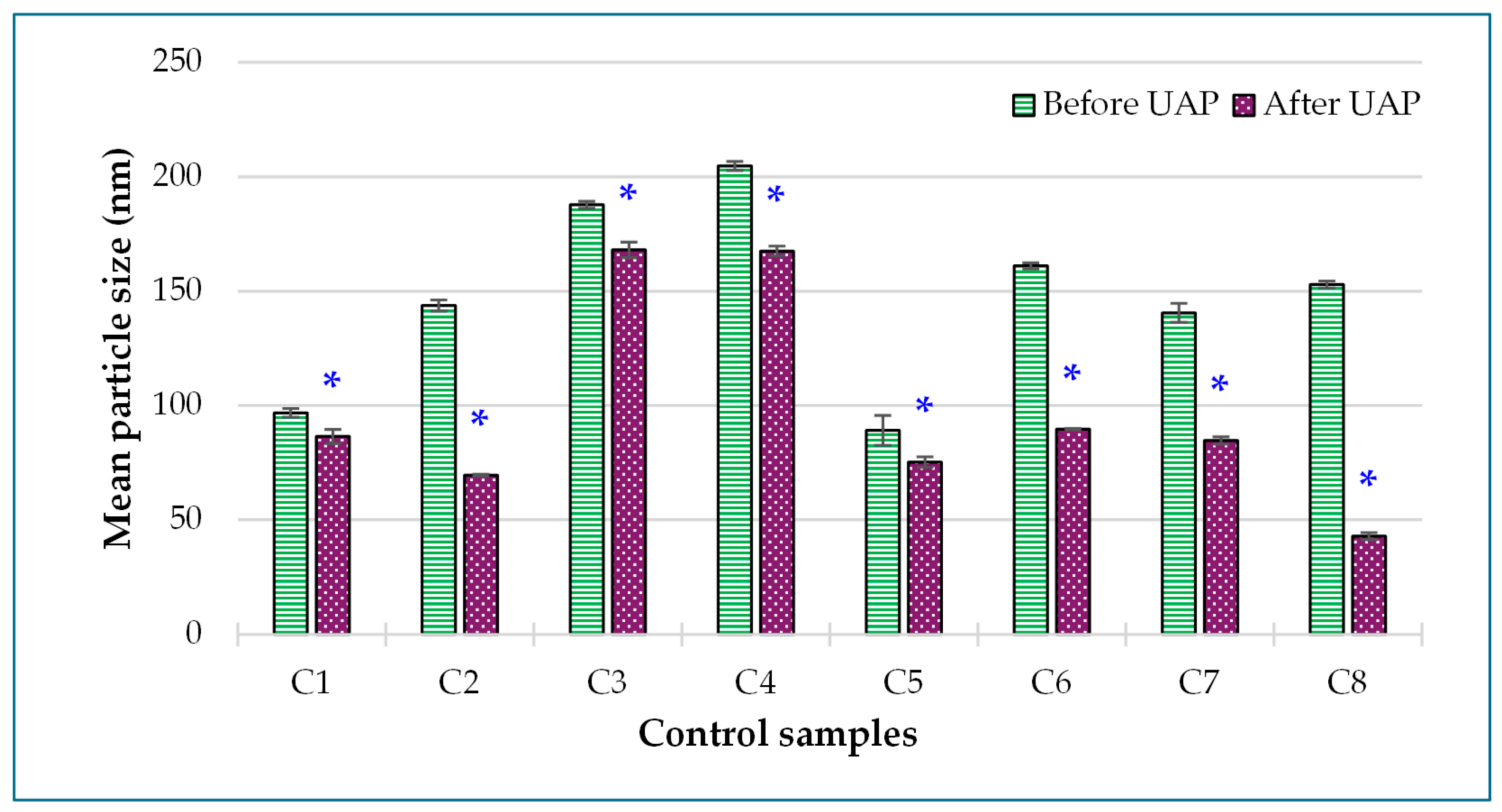
Mean particle size (nm) of control liposomes before and after ultrasound-assisted processing (UAP). Data are presented as mean ± SD (n = 4 independent formulation replicates). * indicates a statistically significant difference between values before and after UAP within the same formulation (paired Student’s *t*-test with Holm adjustment for multiple comparisons, adjusted *p* < 0.05).

Mean particle size before ultrasound bath-assisted processing ranged from 89.0 ± 6.5 nm to 204.7 ± 2.0 nm, depending on phospholipid composition (Figure 2).

Following ultrasound bath-assisted processing, particle size decreased in all formulations, reaching values between 42.9 ± 1.5 nm and 168.0 ± 3.4 nm. The greatest reduction was observed for formulation C8, whereas formulations prepared with Lipoid S75 (C1) and soybean lecithin (C3) exhibited comparatively smaller changes.

**Figure 3:**
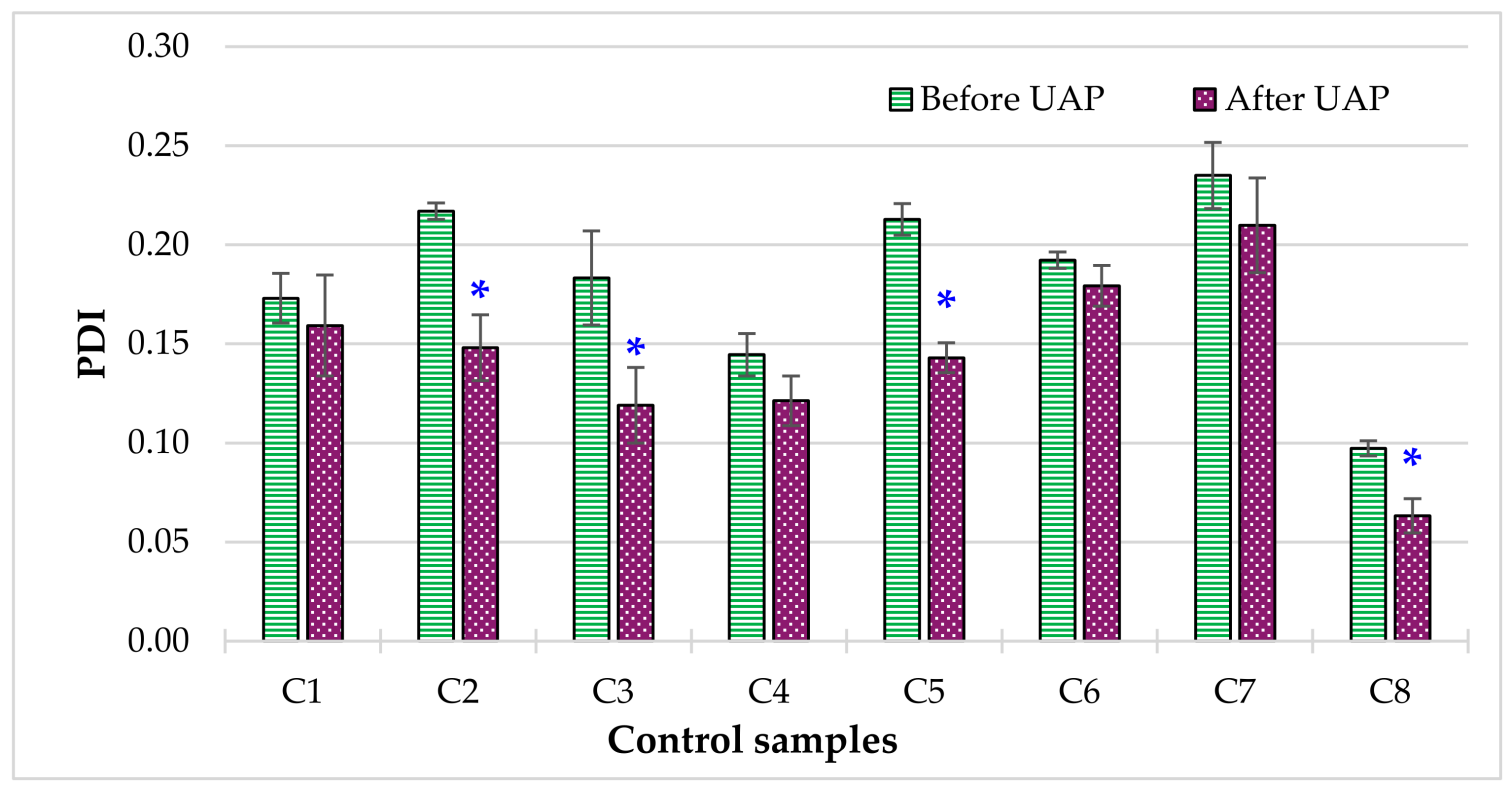
PDI of control liposomes before and after ultrasound-assisted processing (UAP). Data are presented as mean ± SD (n = 4 independent formulation replicates). * indicates a statistically significant difference between values before and after UAP within the same formulation (paired Student’s *t*-test with Holm adjustment for multiple comparisons, adjusted *p* < 0.05).

PDI values remained below 0.30 for all formulations, indicating relatively narrow particle size distributions. UAP resulted in lower PDI values for all phospholipid systems, with statistically significant improvements observed in formulations C2, C3, C5, and C8 (*p* < 0.05) (Figure 3).

**Figure 4:**
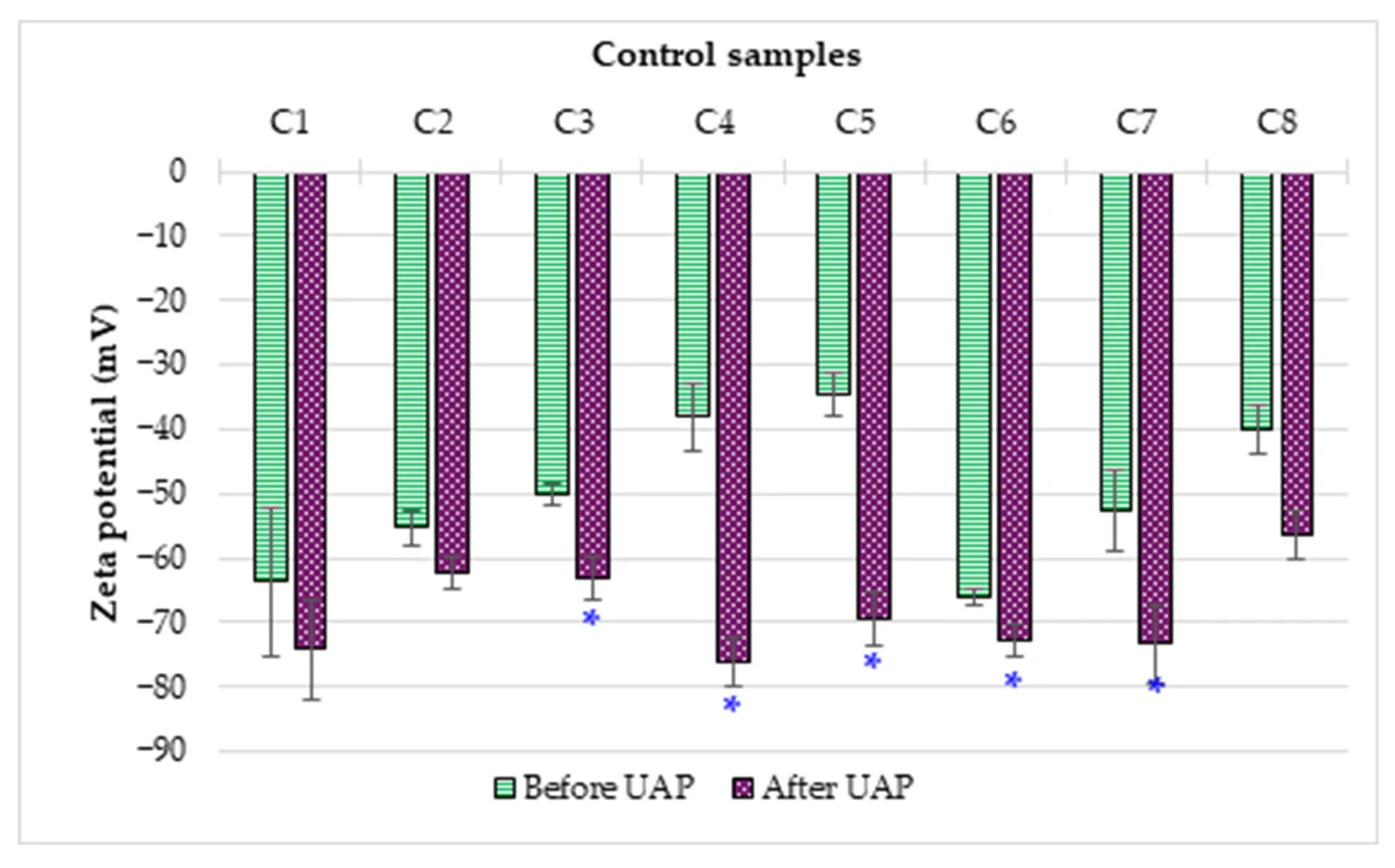
Zeta potential (mV) of control liposomes before and after ultrasound-assisted processing (UAP). Data are presented as mean ± SD (n = 4 independent formulation replicates). * indicates a statistically significant difference between values before and after UAP within the same formulation (paired Student’s *t*-test with Holm adjustment for multiple comparisons, adjusted *p* < 0.05).

All formulations exhibited negative zeta potential values characteristic of phospholipid vesicles. Following ultrasound bath-assisted processing, the absolute zeta potential increased in all formulations, indicating improved electrostatic stabilization of the dispersions (Figure 4).

Overall, phospholipid composition substantially affected the physicochemical characteristics of control liposomes, while UAP generally reduced particle size and PDI and modified zeta potential.

### 3.3. Physicochemical Characterization of Trans-Resveratrol-Containing Liposomes

The influence of phospholipid composition on trans-resveratrol-containing liposomes was evaluated by determining particle size, PDI, and zeta potential.

**Figure 5:**
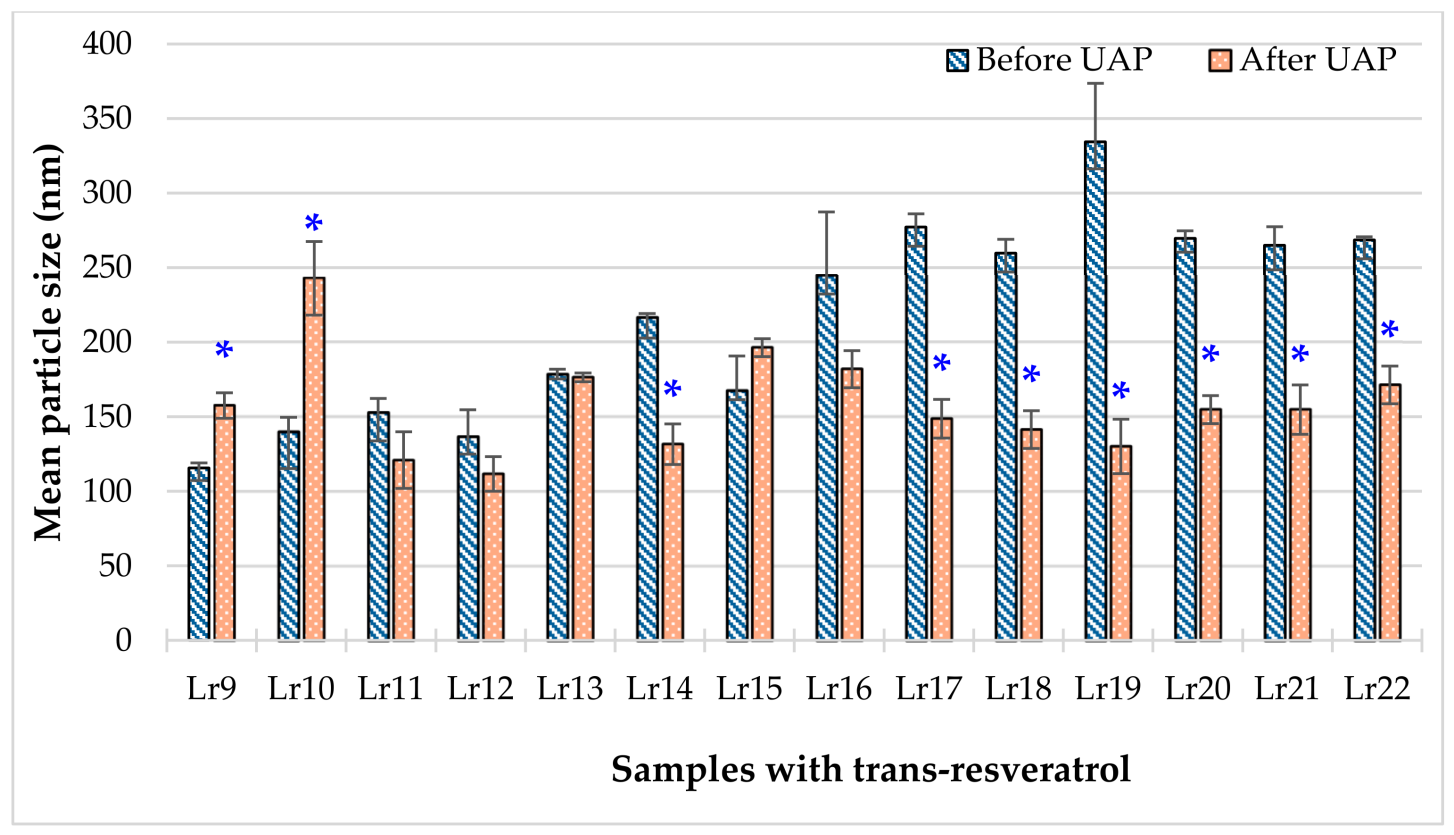
Mean particle size (nm) of trans-resveratrol-containing liposomes before and after ultrasound-assisted processing (UAP). Data are presented as mean ± SD (n = 4 independent formulation replicates). * indicates a statistically significant difference between values before and after UAP within the same formulation (paired Student’s *t*-test with Holm adjustment for multiple comparisons, adjusted *p* < 0.05).

Considerable differences among formulations were observed depending on the phospholipid composition employed. Liposomes prepared with Lipoid P45 exhibited the most favorable physicochemical characteristics, producing nanosized vesicles with relatively narrow particle size distributions (Figure 5).

**Figure 6:**
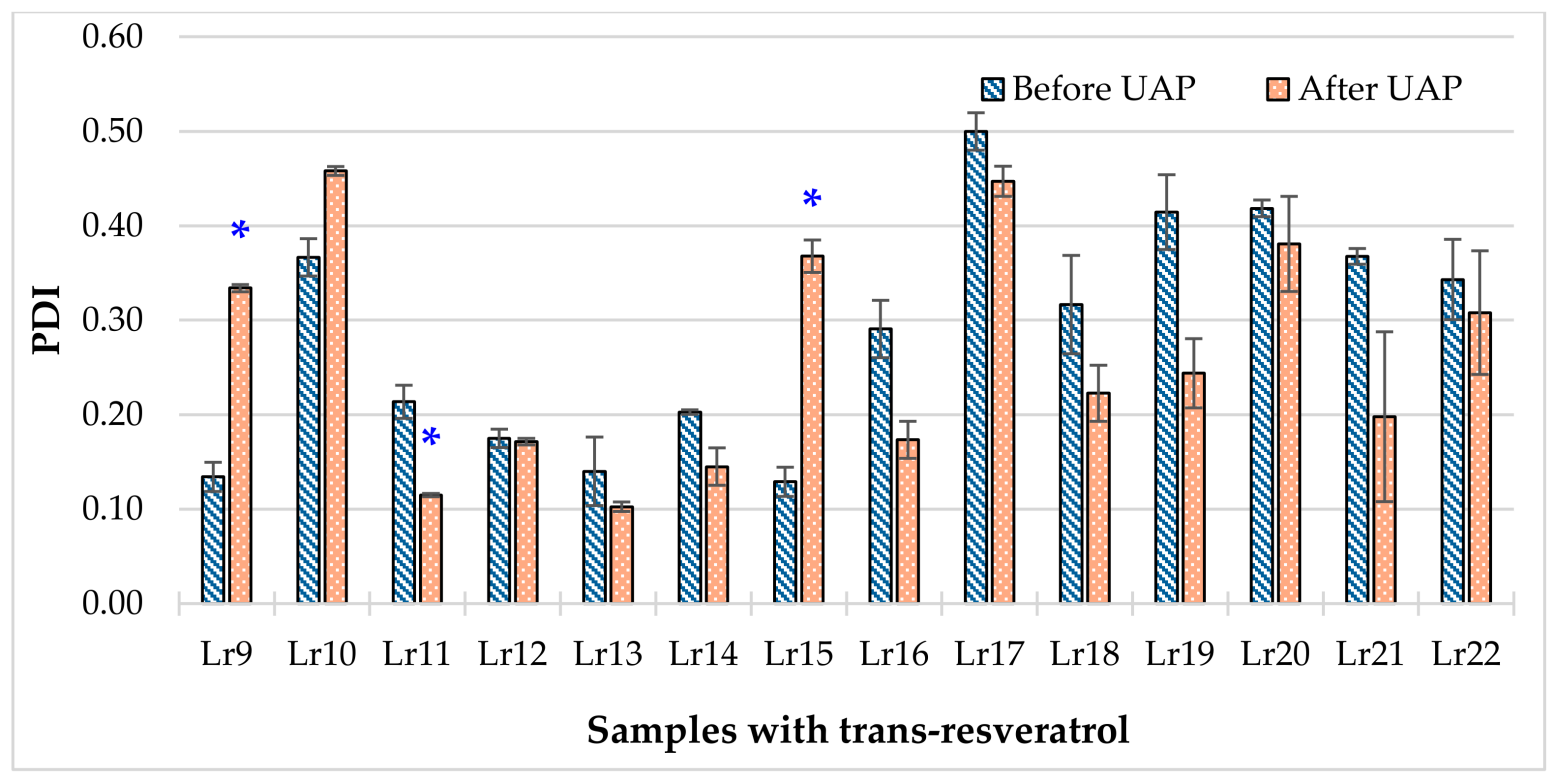
PDI of trans-resveratrol-containing liposomes before and after ultrasound-assisted processing (UAP). Data are presented as mean ± SD (n = 4 independent formulation replicates). * indicates a statistically significant difference between values before and after UAP within the same formulation (paired Student’s *t*-test with Holm adjustment for multiple comparisons, adjusted *p* < 0.05).

For the Lipoid P45-based formulations Lr11 and Lr12, mean particle sizes before UAP were 152.8 ± 9.5 nm and 136.5 ± 18.2 nm, respectively, with corresponding PDI values of 0.214 ± 0.018 and 0.175 ± 0.010 (Figure 5 and Figure 6).

**Figure 7:**
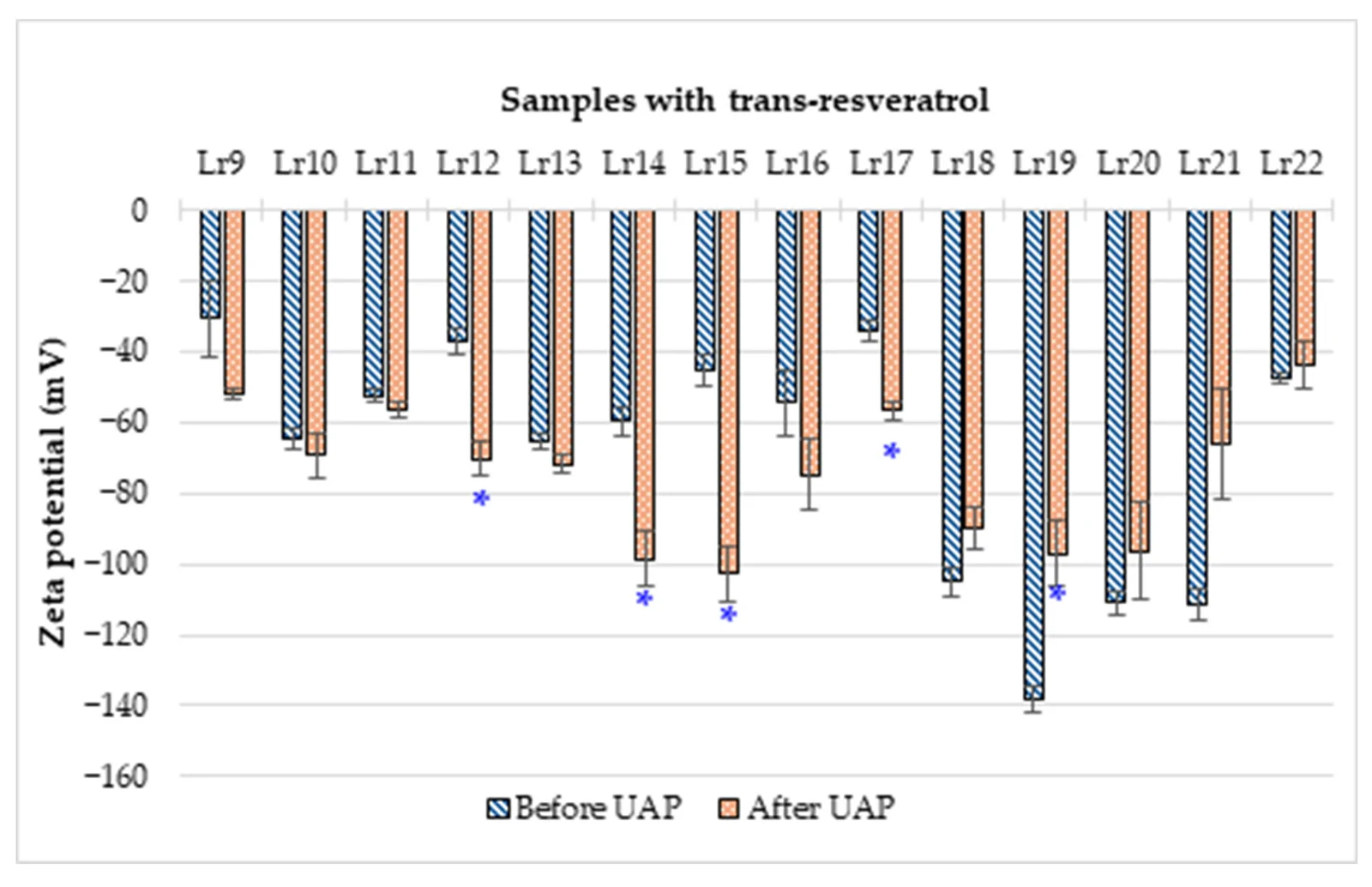
Zeta potential (mV) of trans-resveratrol-containing liposomes before and after ultrasound-assisted processing (UAP). Data are presented as mean ± SD (n = 4 independent formulation replicates). * indicates a statistically significant difference between values before and after UAP within the same formulation (paired Student’s *t*-test with Holm adjustment for multiple comparisons, adjusted *p* < 0.05).

Zeta potential values varied from −36.8 ± 3.7 mV to −52.5 ± 1.9 mV. For Lr18 and Lr19, zeta potential values were strongly negative, indicating substantial electrostatic surface charge (Figure 7).

By contrast, formulations prepared with other phospholipid systems generally exhibited larger particle sizes and broader particle size distributions.

### 3.4. Effect of Ultrasound Bath-Assisted Processing

Ultrasound bath-assisted processing altered the physicochemical characteristics of trans-resveratrol-containing liposomes, with the magnitude of the effect varying among formulations.

Following treatment, particle size and PDI decreased in most trans-resveratrol-containing formulations, although increases in both parameters were observed for Lr9, Lr10, and Lr15. Overall, the magnitude and direction of the UAP response were formulation-dependent.

UAP also modified zeta potential in a formulation-dependent manner. After treatment, zeta potential values ranged from −43.63 ± 6.46 mV to −102.80 ± 7.57 mV, indicating substantial differences in surface charge among formulations.

These findings indicate that UAP generally modified the initial physicochemical characteristics of the liposomal dispersions, although the magnitude and direction of the response depended on phospholipid composition.

### 3.5. Short-Term Physical Stability of Liposomal Formulations During Refrigerated Storage

The short-term physical stability of control and trans-resveratrol-containing liposomal formulations was assessed by comparing mean particle size, PDI, and zeta potential immediately after preparation (day 0) and after 30 days of refrigerated storage. Formulations not subjected to ultrasound-assisted processing (UAP) and those subjected to UAP were evaluated separately.

In the control liposomes, mean particle size generally showed moderate changes during the 30-day storage period (Table 3). In control formulations not subjected to UAP, particle size increased after storage in most samples, although the magnitude of the increase varied among the different phospholipid compositions. Similar formulation-dependent changes were observed in UAP-treated control liposomes. Overall, the control formulations exhibited particle sizes within the nanoscale range after 30 days of storage.

Changes in PDI were also dependent on formulation composition (Table 4). In untreated control liposomes, both increases and decreases in PDI were observed after 30 days. A similar pattern was found in UAP-treated formulations, with some samples showing increased PDI and others showing little change or a decrease. Nevertheless, PDI values remained below 0.30 in all control formulations after 30 days, indicating that pronounced broadening of the particle size distribution did not occur during the investigated storage period.

Zeta potential remained negative in all control formulations throughout storage (Table 5). After 30 days, most control formulations exhibited an increase in the absolute magnitude of the zeta potential compared with the initial measurements. This tendency was observed in both untreated and UAP-treated samples, although the extent of the change differed considerably among the individual formulations.

Trans-resveratrol-containing liposomes exhibited substantially greater formulation-dependent variation during storage than the control liposomes. Changes in mean particle size differed markedly among formulations (Table 6). In several samples, particle size remained comparable to or decreased relative to the initial values, whereas pronounced particle growth was observed in selected formulations. Among the UAP-treated trans-resveratrol-containing liposomes, Lr12, Lr13, and Lr15 showed particularly large increases in mean particle size after 30 days. In contrast, Lr20, Lr21, and Lr22 exhibited comparatively small particle sizes after storage.

A similar formulation-dependent pattern was observed for PDI (Table 7). Several trans-resveratrol-containing formulations showed increased PDI values after 30 days, indicating a broader particle size distribution. Particularly pronounced increases were observed for Lr12, Lr13, and Lr15 following UAP, whereas several other formulations maintained or reached comparatively lower PDI values during storage. Among the UAP-treated formulations, Lr20–Lr22 showed relatively favorable particle size and PDI profiles after 30 days.

The zeta potential of all trans-resveratrol-containing formulations remained negative throughout the storage period (Table 8). However, changes in its magnitude varied substantially among formulations. In some samples, the absolute zeta potential increased after storage, whereas in others it decreased. Importantly, changes in zeta potential did not consistently parallel the changes observed in particle size and PDI; some formulations exhibited strongly negative zeta potential values despite substantial changes in particle size distribution.

Overall, the 30-day short-term physical stability assessment demonstrated that the storage behavior of the liposomal dispersions was strongly dependent on formulation composition and UAP treatment. Control liposomes generally exhibited moderate changes in particle size and PDI, whereas trans-resveratrol-containing formulations showed greater variability. Selected trans-resveratrol-containing formulations exhibited pronounced increases in particle size and polydispersity during storage, while Lr20–Lr22 showed the most favorable physicochemical profiles among the UAP-treated formulations after 30 days.

## 4. Discussion

The present study demonstrates that the physicochemical behavior of trans-resveratrol-containing liposomes prepared by the ethanol injection method is governed by the combined effects of phospholipid composition, the nominal amount of trans-resveratrol added during formulation, ultrasound-assisted processing (UAP), and storage. The inclusion of corresponding blank liposomal formulations was particularly informative because it enabled the effects associated with the presence of trans-resveratrol in the formulation.

The results showed that phospholipid composition strongly influenced particle size, PDI, and zeta potential at initial characterization, whereas the behavior of the trans-resveratrol-containing systems became considerably more formulation-dependent during storage.

A pronounced effect of phospholipid composition was already evident in the blank formulations. Although all investigated lipid systems produced nanoscale vesicles, the resulting particle size, size distribution, and surface charge differed considerably. This finding is consistent with previous studies demonstrating that phospholipid source, fatty acid saturation, membrane packing, and phase behavior have a direct influence on vesicle formation and storage performance. Song et al. reported substantial differences between soybean phosphatidylcholine-, egg yolk phosphatidylcholine-, and hydrogenated soybean phosphatidylcholine-based liposomes, showing that phospholipid composition affected vesicle size, membrane fluidity, packing density, and long-term stability [23]. More recent work has similarly emphasized that formulation composition and preparation conditions are among the principal determinants of liposomal stability throughout production and storage [24].

These observations provide a plausible basis for the differences found among Lipoid^®^ S75, Lipoid^®^ P45, soybean lecithin, egg yolk lecithin, and their binary mixtures in the present study. Differences in phosphatidylcholine content and fatty acid composition can modify membrane fluidity, curvature, mechanical resistance, and susceptibility to oxidation or hydrolysis. Therefore, the phospholipid source should not be regarded merely as an interchangeable excipient but rather as a critical formulation variable controlling the physicochemical characteristics of the resulting liposomes.

The phospholipid materials used in this study differed substantially in phosphatidylcholine (PC) content and fatty acid composition, which may have contributed to the formulation-dependent physicochemical behavior. The soybean-derived phospholipids were characterized by a high proportion of unsaturated fatty acids, particularly linoleic acid, whereas egg yolk phosphatidylcholine showed a more balanced distribution of saturated and unsaturated fatty acids. Differences in fatty acid saturation and PC content may influence lipid packing, membrane fluidity, and interfacial properties and could therefore contribute to the observed differences in particle size, PDI, zeta potential, and responses to UAP and refrigerated storage.

The influence of PC content should, however, not be considered independently of phospholipid source and fatty acid composition, because materials with different PC percentages also contain different proportions of other phospholipid and lipid components. Previous studies have demonstrated that phospholipid composition and fatty acid saturation can influence vesicle size, size distribution, membrane packing, fluidity, and surface properties [19,23,24]. In particular, Song et al. [23] reported distinct particle sizes and membrane characteristics for liposomes prepared from soybean, egg yolk, and hydrogenated soybean phosphatidylcholines, with these differences being associated with fatty acid composition and degree of saturation. Thus, the differences observed among the present formulations likely reflect the combined effects of PC content, fatty acid composition, and accompanying lipid components rather than PC percentage alone. Because the fatty acid composition of the individual experimental batches and the structural organization of the lipid bilayers were not directly determined in the present study, these relationships should be regarded as literature- and manufacturer-data-supported hypotheses rather than experimentally demonstrated mechanisms.

Cholesterol was intentionally not included in the present formulations. Although cholesterol is commonly incorporated into liposomal membranes to modulate bilayer rigidity, permeability, and physical stability, its inclusion would have introduced an additional membrane-modifying variable. Since the primary objective of this study was to compare the influence of phospholipid source and composition under otherwise comparable preparation conditions, omission of cholesterol enabled the effects associated specifically with the investigated phospholipid systems to be evaluated more directly. Nevertheless, cholesterol represents an important formulation variable, and its influence on the physicochemical characteristics and short-term physical stability of the selected formulations should be investigated in future optimization studies.

The effect of lipid composition became more complex in the presence of trans-resveratrol. The trans-resveratrol-containing formulations displayed a broader range of particle sizes and PDI values than the corresponding blank systems. This observation suggests that the presence of trans-resveratrol during formulation may have influenced the physicochemical organization of the lipid dispersions. This interpretation is consistent with previous membrane-model studies showing that resveratrol can interact with phosphatidylcholine bilayers; however, the extent of its incorporation into the liposomal systems investigated here was not determined. Vitkova et al. demonstrated that resveratrol associates spontaneously with phosphatidylcholine bilayers and that the extent and consequences of this interaction depend on the degree of glycerophospholipid unsaturation [25]. In monounsaturated bilayers, resveratrol behaved as a molecular “filler” that increased lipid packing, whereas in more highly unsaturated membranes it acted more like a “spacer”, reducing lipid order [25]. Earlier membrane-model studies also showed that resveratrol can directly incorporate into phosphatidylcholine-containing lipid bilayers and modify their biophysical properties [26].

This interaction may explain why corresponding blank and trans-resveratrol-containing formulations did not always behave similarly. Because the investigated phospholipid materials differ in composition and degree of unsaturation, the presence of trans-resveratrol during formulation may have altered bilayer organization to different extents. A formulation that forms a relatively stable blank liposome may therefore respond differently in the presence of trans-resveratrol. This finding is particularly relevant for natural phospholipid systems, in which the bilayer consists of heterogeneous lipid species rather than a single chemically defined phosphatidylcholine.

The nominal amount of trans-resveratrol also appeared to influence formulation behavior. Liposomes prepared with the same phospholipid system but different trans-resveratrol amounts did not necessarily exhibit equivalent particle size or storage stability. This suggests that the lipid-to-compound ratio may influence bilayer packing and vesicle organization. However, because encapsulation efficiency and the amount of non-encapsulated trans-resveratrol were not quantified in the present study, this relationship cannot be interpreted as a direct dose-dependent effect. Instead, the results indicate that the nominal amount of trans-resveratrol added during formulation should be considered together with lipid composition rather than interpreted as an independent drug-loading parameter. Accordingly, differences between formulations containing nominally 100 and 200 mg of trans-resveratrol cannot be interpreted as dose-dependent effects of the amount actually incorporated into the liposomal bilayer.

UAP exerted a clear immediate effect on the physicochemical properties of the liposomal dispersions. In the blank formulations, particle size decreased after treatment in all investigated phospholipid systems, accompanied by generally reduced PDI values. Similar size-reduction effects were observed in many of the trans-resveratrol-containing formulations, although their magnitude depended on composition. The observed reduction is consistent with the established effect of ultrasound on liposomal dispersions, where cavitation and associated shear forces disrupt larger vesicles and generate smaller populations.

Yun et al. compared different ultrasonic approaches for liposome preparation and demonstrated that ultrasound-based processing can substantially reduce particle size and produce narrower size distributions [27]. Their work also confirmed that bath-type sonication is capable of modifying liposome size, although more focused ultrasonic energy provides tighter size control. Similarly, Baek et al. produced resveratrol-loaded liposomes of approximately 300, 150, and 75 nm by increasing ultrasonication time, demonstrating a direct relationship between ultrasonic treatment and particle size reduction [28]. These findings support the present observation that even a conventional ultrasonic bath can serve as a practical post-formulation size-reduction step.

Nevertheless, the present data also demonstrate an important distinction between immediate particle size reduction and short-term physical storage behavior. Although UAP improved the initial physicochemical properties of many formulations, the same advantage was not uniformly exhibited during 30 days of storage. Therefore, the effect of an ultrasonic bath should be considered primarily as a formulation-dependent post-processing intervention rather than as an inherently stabilizing treatment.

This distinction is supported by the study of Baek et al., who showed that reducing the size of resveratrol-loaded liposomes improved solubility, release behavior, cellular permeability, and oral bioavailability, but did not improve antioxidant stability. The authors attributed this partly to the larger surface area of smaller liposomes, which may increase their interaction with the surrounding environment [28]. Thus, smaller particle size is not necessarily synonymous with superior stability, and storage behavior must be evaluated independently from their initial particle size characteristics.

PDI provided additional information that could not be obtained from particle size alone. In the control formulations, PDI values remained below 0.30 throughout the investigated storage period, suggesting that marked broadening of the size distribution did not occur. In contrast, several trans-resveratrol-containing formulations showed substantial increases in PDI during storage. High PDI values, particularly when accompanied by large mean hydrodynamic diameters and high standard deviations, indicate the development of heterogeneous particle populations rather than uniform enlargement of the entire vesicle population. Accordingly, the pronounced increases observed in selected formulations may reflect physical destabilization associated with aggregation, vesicle fusion, restructuring, or coexistence of multiple particle populations.

The 30-day refrigerated storage study provided particularly important evidence of this formulation-dependent behavior. Blank liposomes generally exhibited moderate changes in particle size and PDI, whereas the trans-resveratrol-containing systems showed substantially greater variability. Among the UAP-treated trans-resveratrol-containing formulations, Lr12, Lr13, and Lr15 showed marked particle growth after 30 days, reaching approximately 513, 431, and 524 nm, respectively, while their PDI values increased to approximately 0.51–0.56. The simultaneous increase in particle size and PDI suggests pronounced physical instability of these systems.

The behavior of Lr12 is particularly informative. Although Lipoid^®^ P45-based formulations exhibited favorable initial characteristics, LR12 did not retain this advantage during storage. This demonstrates that initial particle size and PDI alone are insufficient to define an “optimized” formulation. The short-term physical storage behavior of a trans-resveratrol-containing liposomal system can differ markedly from that predicted from its day-0 characterization. Accordingly, formulation selection should include both initial physicochemical screening and subsequent storage evaluation.

In contrast, Lr20, Lr21, and Lr22 exhibited the most favorable physicochemical profiles among UAP-treated formulations after storage. Their particle sizes after 30 days were approximately 57, 56, and 67 nm, respectively, while PDI values were approximately 0.195, 0.157, and 0.131. These formulations contained binary phospholipid systems: soybean lecithin/egg yolk lecithin for Lr20 and Lipoid^®^ P45/egg yolk lecithin for Lr21–Lr22.

The favorable behavior of these binary systems may reflect complementary effects of their lipid components on membrane organization. Combining phospholipids with different fatty acid profiles and packing properties may provide a better balance between membrane flexibility and mechanical resistance than selected single-phospholipid systems. Evidence that lipid composition can markedly alter membrane fluidity, packing, and storage stability supports this interpretation [23,24]. However, this mechanism cannot be established from DLS and zeta potential measurements alone and should therefore be presented as a plausible explanation rather than a demonstrated molecular mechanism.

Another noteworthy feature was that Lr20–Lr22 became substantially smaller during storage compared with their day-0 UAP-treated values. This behavior should not simply be described as absence of change or conventional physical stability. Instead, it may indicate continued reorganization of the dispersed lipid system, including redistribution of vesicle populations or restructuring of the bilayer. Because morphology and lamellarity were not directly investigated, the mechanism underlying this decrease cannot be resolved from the present data. Nevertheless, the simultaneous retention of comparatively low PDI values indicates that these dispersions maintained favorable colloidal characteristics after 30 days.

The present findings can also be compared with other resveratrol liposome studies. Zhang et al. reported resveratrol nanoliposomes with a mean particle size of approximately 56 nm, PDI of approximately 0.15, and good stability at 4 °C for 90 days, during which only a small increase in particle size was observed [29]. Although the formulation composition and preparation method differed from those used in the present study, these results confirm that appropriately formulated resveratrol-loaded liposomes can maintain small particle sizes and narrow distributions during refrigerated storage. The contrasting behavior of Lr12, Lr13, and Lr15 further emphasizes that this stability is strongly composition-dependent rather than an intrinsic property of resveratrol liposomes.

Zeta potential provided an additional but not independently sufficient indicator of colloidal behavior. All investigated formulations exhibited negative zeta potential values, yet strongly negative values did not always correspond to small particle size or low PDI after storage. This is an important observation because zeta potential is frequently interpreted as a direct indicator of colloidal stability. Although higher absolute surface charge can enhance electrostatic repulsion, stability also depends on membrane packing, hydration, steric effects, ionic environment, and chemical changes occurring during storage. The 2025 review by Xu et al. similarly emphasized that liposomal stability results from the combined effects of formulation, processing, storage environment, and interfacial phenomena rather than from a single physicochemical parameter [24].

Accordingly, particle size, PDI, and zeta potential should be interpreted collectively. In the present study, formulations with large absolute zeta potentials but simultaneously increased particle size and PDI cannot be considered physically stable solely on the basis of electrostatic surface charge. This multidimensional interpretation provides a more reliable assessment of storage behavior.

A further practical implication of the present results is that formulation screening should not rely exclusively on day-0 measurements. Initially favorable particle size and PDI values can identify technically promising systems, but they do not necessarily predict storage performance. The results therefore support a two-stage formulation strategy: initial selection based on particle size, PDI, and zeta potential, followed by confirmation of stability during storage. This approach is particularly important for trans-resveratrol because interactions between the compound and the lipid bilayer can alter the properties of the final trans-resveratrol-containing system relative to the corresponding blank carrier.

The use of a conventional ultrasonic bath represents another practically relevant aspect of the study. Many liposome-size reduction methods rely on probe sonication, extrusion, high-pressure homogenization, or other specialized equipment. In contrast, the present approach used an inexpensive and widely available ultrasonic bath. Although the energy input is lower and less controlled than in focused or probe-based systems, the observed immediate reduction in particle size demonstrates that conventional bath-assisted processing can still provide meaningful physicochemical modification. The practical value of this method lies therefore in its accessibility rather than in achieving the highly controlled energy delivery associated with more advanced ultrasonic technologies.

The present study has several limitations that should be considered when interpreting the findings. The stability assessment was limited to physicochemical parameters over a 30-day refrigerated storage period; therefore, the results describe short-term physical stability rather than long-term pharmaceutical stability. Chemical stability of trans-resveratrol was not assessed, as trans-resveratrol retention or degradation during storage was not quantitatively determined. In addition, although the identity of trans-resveratrol used for formulation was confirmed by LC–MS, its quantitative retention within the formulations during storage was not evaluated. Because no direct structural or molecular-level characterization was performed, the proposed explanations involving changes in membrane organization, bilayer restructuring, molecular filler/spacer effects, or vesicle reorganization should be regarded as literature-supported hypotheses rather than mechanisms demonstrated by the present study. Further studies combining longer-term stability testing with quantitative analysis of trans-resveratrol and complementary structural characterization would be valuable to confirm the performance of the most promising formulations.

Overall, the findings demonstrate that the physicochemical performance of trans-resveratrol-containing liposomes cannot be attributed to a single formulation parameter. Phospholipid composition determined the initial properties of the lipid vesicles, the presence of trans-resveratrol was associated with formulation-dependent changes in physicochemical behavior, and UAP effectively reduced particle size in many freshly prepared dispersions. However, the 30-day refrigerated storage study showed that the effects of these factors on storage behavior were strongly formulation-dependent. Selected formulations, particularly Lr12, Lr13, and Lr15, displayed pronounced physical destabilization, whereas Lr20–Lr22 exhibited the most favorable particle size and PDI profiles among the UAP-treated systems. These findings emphasize that lipid composition, the nominal amount of trans-resveratrol added during formulation, post-processing, and storage behavior must be evaluated together when selecting a suitable liposomal formulation. Encapsulation efficiency and actual trans-resveratrol loading were not quantitatively determined. Consequently, the nominal amounts of 100 and 200 mg added during formulation cannot be considered equivalent to the amounts incorporated into the liposomal system, and formulation differences cannot be interpreted as direct dose-dependent effects of incorporated trans-resveratrol.

## 5. Conclusions

The physicochemical performance of trans-resveratrol-containing liposomes was strongly dependent on phospholipid composition, the nominal amount of trans-resveratrol added during formulation, and post-formulation UAP. The ethanol injection method enabled the preparation of nanoscale liposomal dispersions using different natural phospholipid systems, while a conventional ultrasonic bath provided a simple and accessible means of reducing particle size and, in many formulations, improving particle size distribution immediately after preparation.

However, the 30-day refrigerated storage study demonstrated that the beneficial initial effect of ultrasound-assisted processing did not uniformly translate into improved short-term physical stability. Storage behavior was formulation-dependent, with selected trans-resveratrol-containing formulations showing pronounced particle growth and increased polydispersity, whereas Lr20–Lr22 exhibited the most favorable particle size and PDI profiles after refrigerated storage.

Overall, the results emphasize that liposome development should not rely solely on initial particle size reduction or zeta potential values. Phospholipid composition, the nominal amount of trans-resveratrol added during formulation, post-processing response, and storage behavior should be evaluated together when selecting a suitable formulation. The favorable performance of selected binary phospholipid formulations, particularly Lr20–Lr22, identifies these formulations as promising candidates for further development.

Future studies should include encapsulation efficiency, quantitative assessment of trans-resveratrol stability during storage, vesicle morphology, release behavior, and longer-term physical and chemical stability testing to assess the pharmaceutical potential of the most promising formulations.

## Figures and Tables

**Table 1:** Composition and manufacturer specifications of the phospholipid materials used for liposome preparation.

Phospholipid Material	Biological Source	Phosphatidylcholine (PC) Content	Typical/Reported Fatty Acid Composition (%)	Degree of Saturation *	Purity/Product Specification
Lipoid S75	Soybean	≥70% PC	C16:0 15%; C18:0 3%; C18:1 12%; C18:2 62%; C18:3 5% **	Predominantly unsaturated; approx. 18% saturated/79% unsaturated	Commercial soybean lecithin; separate overall purity not specified
Lipoid P45	Non-GMO soybean	≥45% PC	C16:0 15%; C18:0 3%; C18:1 12%; C18:2 62%; C18:3 5% **	Predominantly unsaturated; approx. 18% saturated/79% unsaturated	Commercial non-GMO soybean phospholipid/lecithin; separate overall purity not specified
L-α-Phosphatidylcholine, Type IV-S	Soybean	≥30% (enzymatic); manufacturer data for selected lots report ~55% PC on average (42–63%, TLC)	C16:0 17%; C18:0 4%; C18:1 9%; C18:2 60%; C18:3 7% *	Predominantly unsaturated; approx. 21% saturated/76% unsaturated	Type IV-S; ≥30% (enzymatic); natural phospholipid mixture; separate overall purity not specified
L-α-Phosphatidylcholine	Egg yolk	~60% (TLC)	C16:0 ~33%; C18:0 ~13%; C18:1 ~31%; C18:2 ~15% **	Mixed profile; approx. 46% saturated/46% unsaturated	~60% PC by TLC; natural egg yolk phospholipid mixture; separate overall purity not specified

* Degree of saturation was estimated from the reported major fatty acid composition by summing saturated (C16:0 and C18:0) and unsaturated fatty acids (C18:1, C18:2, C18:3, and other reported unsaturated species, where applicable). ** Fatty acid compositions reported for Lipoid S75 and P45 represent typical source-level profiles for natural soybean phosphatidylcholine provided by the manufacturer and should not be interpreted as batch-specific analyses of the materials used in this study. The fatty acid composition shown for Sigma-Aldrich 61755 represents the typical profile of egg yolk phosphatidylcholine reported by the manufacturer and is likewise not batch-specific. For Sigma-Aldrich P3644, the fatty acid values correspond to selected lots characterized by the manufacturer. Minor fatty acids not individually listed account for the remaining percentage. PC, phosphatidylcholine.

**Table 2:** Composition of control and trans-resveratrol-containing liposomal formulations.

Lipid Composition	Phospholipid Amount (mg)	Control	+100 mg Trans-Resveratrol	+200 mg Trans-Resveratrol
Lipoid S75	300	C1	Lr9	Lr10
Lipoid P45	300	C2	Lr11	Lr12
Soy lecithin	300	C3	Lr13	Lr14
Egg yolk lecithin	300	C4	Lr15	Lr16
Lipoid S75/Lipoid P45	150/150	C5	Lr17	Lr18
Soy lecithin/egg yolk lecithin	150/150	C6	Lr19	Lr20
Lipoid S75/soy lecithin	150/150	C7	—	—
Lipoid P45/egg yolk lecithin	150/150	C8	Lr21	Lr22

C—control samples without trans-resveratrol, Lr—samples with trans-resveratrol. Control formulations were included to distinguish the influence of phospholipid composition from that associated with the presence of trans-resveratrol in the formulation. The amounts of trans-resveratrol refer to the nominal quantities added during formulation preparation; encapsulation efficiency and actual drug loading were not determined.

**Table 3:** Mean particle size (nm) of control liposomal formulations before and after ultrasound-assisted processing at day 0 and after 30 days of refrigerated storage.

Control Samples	Day 0		Day 30	
	Before UAP	After UAP	Before UAP	After UAP
C1	96.64 ± 1.90	86.35 ± 3.10 *	117.93 ± 0.61 #	111.30 ± 4.30 #
C2	143.75 ± 2.43	69.47 ± 0.41 *	157.08 ± 1.14 #	86.15 ± 5.47 *
C3	187.73 ± 1.52	168.03 ± 3.41 *	196.73 ± 7.72	181.10 ± 7.70
C4	204.70 ± 1.98	167.53 ± 2.17 *	226.08 ± 1.93 #	162.53 ± 4.48 *
C5	89.00 ± 6.52	75.17 ± 2.30 *	106.28 ± 1.92	80.22 ± 2.14 *#
C6	161.05 ± 1.31	89.56 ± 0.31 *	174.33 ± 3.60 #	94.51 ± 3.13 *
C7	140.55 ± 4.16	84.60 ± 1.61 *	158.98 ± 2.96 #	97.84 ± 5.16 *#
C8	152.93 ± 1.53	42.86 ± 1.54 *	153.93 ± 2.18	77.40 ± 7.68 *

Data are presented as mean ± SD (n = 4 independent formulation replicates). * indicates a significant difference from the corresponding value before UAP at the same storage time; # indicates a significant difference from the corresponding value at day 0 under the same UAP condition (paired Student’s *t*-test with Holm adjustment for multiple comparisons, adjusted *p* < 0.05).

**Table 4:** PDI of control liposomal formulations before and after ultrasound-assisted processing at day 0 and after 30 days of refrigerated storage.

Control Samples	Day 0		Day 30	
	Before UAP	After UAP	Before UAP	After UAP
C1	0.17 ± 0.01	0.16 ± 0.02	0.17 ± 0.02	0.16 ± 0.02
C2	0.22 ± 0.01	0.15 ± 0.02 *	0.20 ± 0.02	0.14 ± 0.02
C3	0.18 ± 0.02	0.12 ± 0.02 *	0.20 ± 0.03	0.22 ± 0.01
C4	0.14 ± 0.01	0.12 ± 0.01	0.22 ± 0.02 #	0.25 ± 0.01
C5	0.21 ± 0.01	0.14 ± 0.01 *	0.21 ± 0.02	0.19 ± 0.02 #
C6	0.19 ± 0.01	0.18 ± 0.01	0.09 ± 0.02 #	0.19 ± 0.01
C7	0.24 ± 0.02	0.21 ± 0.02	0.20 ± 0.01	0.13 ± 0.01
C8	0.10 ± 0.01	0.06 ± 0.01 *	0.14 ± 0.02	0.12 ± 0.02

Data are presented as mean ± SD (n = 4 independent formulation replicates). * indicates a significant difference from the corresponding value before UAP at the same storage time; # indicates a significant difference from the corresponding value at day 0 under the same UAP condition (paired Student’s *t*-test with Holm adjustment for multiple comparisons, adjusted *p* < 0.05).

**Table 5:** Zeta potential (mV) of control liposomal formulations before and after ultrasound-assisted processing at day 0 and after 30 days of refrigerated storage.

Control Samples	Day 0		Day 30	
	Before UAP	After UAP	Before UAP	After UAP
C1	−63.75 ± 3.71	−74.20 ± 7.67	−51.68 ± 4.26	−66.30 ± 1.98
C2	−55.25 ± 2.75	−62.25 ± 2.36	−135.75 ± 4.01 #	−162.75 ± 4.91 *#
C3	−50.25 ± 1.71	−63.00 ± 3.37 *	−142.00 ± 5.42 #	−155.75 ± 6.79 #
C4	−38.08 ± 5.22	−76.25 ± 3.77 *	−177.00 ± 3.49 #	−131.55 ± 6.99
C5	−34.50 ± 3.42	−69.43 ± 4.28 *	−76.43 ± 3.89 #	−93.73 ± 6.91
C6	−66.08 ± 1.13	−72.75 ± 2.50 *	−147.50 ± 5.29 #	−132.50 ± 5.49 #
C7	−52.70 ± 3.21	−73.40 ± 5.99 *	−85.40 ± 7.27 #	−91.43 ± 6.43
C8	−40.00 ± 3.74	−56.50 ± 3.69	−145.25 ± 4.29 #	−145.28 ± 7.60

Data are presented as mean ± SD (n = 4 independent formulation replicates). * indicates a significant difference from the corresponding value before UAP at the same storage time; # indicates a significant difference from the corresponding value at day 0 under the same UAP condition (paired Student’s *t*-test with Holm adjustment for multiple comparisons, adjusted *p* < 0.05).

**Table 6:** Mean particle size (nm) of trans-resveratrol-containing liposomal formulations before and after ultrasound-assisted processing at day 0 and after 30 days of refrigerated storage.

Samples with Trans-Resveratrol	Day 0		Day 30	
	Before UAP	After UAP	Before UAP	After UAP
Lr9	115.80 ± 3.35	157.50 ± 8.53 *	152.85 ± 4.84 #	186.95 ± 12.88
Lr10	139.88 ± 9.76	242.85 ± 24.68 *	166.55 ± 3.12 #	191.20 ± 10.25
Lr11	152.78 ± 9.54	120.85 ± 18.99	116.20 ± 2.68 #	169.45 ± 40.94
Lr12	136.50 ± 18.19	111.75 ± 11.61	305.45 ± 62.14	512.93 ± 501.23
Lr13	178.25 ± 3.72	176.40 ± 2.95	194.60 ± 6.53	431.00 ± 90.86
Lr14	216.30 ± 2.94	131.53 ± 13.61 *	244.48 ± 21.35	204.23 ± 7.43 #
Lr15	167.35 ± 23.31	196.33 ± 5.94	277.45 ± 89.03	523.63 ± 261.90
Lr16	244.70 ± 42.59	181.90 ± 12.46	238.77 ± 69.33	144.18 ± 2.06
Lr17	277.23 ± 8.95	148.73 ± 12.92 *	147.95 ± 15.58 #	154.25 ± 7.20
Lr18	259.80 ± 9.17	141.35 ± 12.69 *	120.12 ± 4.58 #	112.70 ± 3.84
Lr19	334.50 ± 39.11	130.08 ± 18.26 *	165.93 ± 3.01 #	86.94 ± 1.81 *
Lr20	269.73 ± 4.87	154.88 ± 9.39 *	213.63 ± 41.41	56.76 ± 0.44 #
Lr21	265.03 ± 12.42	154.85 ± 16.58 *	154.60 ± 2.17 #	56.04 ± 0.73 *#
Lr22	268.60 ± 2.19	171.35 ± 12.69 *	111.28 ± 1.55 #	67.00 ± 1.01 *#

Data are presented as mean ± SD (n = 4 independent formulation replicates). * indicates a significant difference from the corresponding value before UAP at the same storage time; # indicates a significant difference from the corresponding value at day 0 under the same UAP condition (paired Student’s *t*-test with Holm adjustment for multiple comparisons, adjusted *p* < 0.05).

**Table 7:** PDI of trans-resveratrol-containing liposomal formulations before and after ultrasound-assisted processing at day 0 and after 30 days of refrigerated storage.

Samples with Trans-Resveratrol	Day 0		Day 30	
	Before UAP	After UAP	Before UAP	After UAP
Lr9	0.134 ± 0.016	0.334 ± 0.004 *	0.117 ± 0.007	0.296 ± 0.067
Lr10	0.367 ± 0.020	0.458 ± 0.005	0.093 ± 0.047 #	0.099 ± 0.056 #
Lr11	0.214 ± 0.018	0.115 ± 0.002 *	0.109 ± 0.022 #	0.243 ± 0.107
Lr12	0.175 ± 0.010	0.172 ± 0.003	0.361 ± 0.037 #	0.510 ± 0.320
Lr13	0.140 ± 0.036	0.103 ± 0.005	0.155 ± 0.020	0.555 ± 0.241
Lr14	0.203 ± 0.002	0.145 ± 0.020	0.619 ± 0.069 #	0.175 ± 0.040 *
Lr15	0.129 ± 0.016	0.368 ± 0.017 *	0.211 ± 0.102	0.535 ± 0.191
Lr16	0.291 ± 0.031	0.174 ± 0.020	0.190 ± 0.091	0.258 ± 0.007
Lr17	0.500 ± 0.020	0.447 ± 0.016	0.184 ± 0.031 #	0.131 ± 0.044 #
Lr18	0.317 ± 0.052	0.223 ± 0.030	0.174 ± 0.014 #	0.161 ± 0.049
Lr19	0.414 ± 0.040	0.244 ± 0.037	0.155 ± 0.022 #	0.246 ± 0.006 *
Lr20	0.419 ± 0.009	0.381 ± 0.050	0.325 ± 0.104	0.195 ± 0.006
Lr21	0.368 ± 0.008	0.198 ± 0.090	0.248 ± 0.024 #	0.157 ± 0.017 *
Lr22	0.343 ± 0.042	0.308 ± 0.065	0.138 ± 0.033 #	0.131 ± 0.014

Data are presented as mean ± SD (n = 4 independent formulation replicates). * indicates a significant difference from the corresponding value before UAP at the same storage time; # indicates a significant difference from the corresponding value at day 0 under the same UAP condition (paired Student’s *t*-test with Holm adjustment for multiple comparisons, adjusted *p* < 0.05).

**Table 8:** Zeta potential (mV) of trans-resveratrol-containing liposomal formulations before and after ultrasound-assisted processing at day 0 and after 30 days of refrigerated storage.

Samples with Trans-Resveratrol	Day 0		Day 30	
	Before UAP	After UAP	Before UAP	After UAP
Lr9	−30.48 ± 10.62	−51.85 ± 1.38	−36.20 ± 7.78	−61.55 ± 5.94 *
Lr10	−64.65 ± 2.86	−69.33 ± 6.20	−51.23 ± 2.65	−55.50 ± 2.21
Lr11	−52.50 ± 1.91	−56.28 ± 2.11	−85.95 ± 5.54 #	−98.85 ± 10.18
Lr12	−36.78 ± 3.71	−70.33 ± 4.78 *	−105.00 ± 4.08 #	−97.70 ± 15.77
Lr13	−65.03 ± 2.19	−71.83 ± 2.57	−85.60 ± 5.89	−85.40 ± 17.29
Lr14	−59.65 ± 3.91	−98.43 ± 7.78 *	−115.75 ± 10.69 #	−111.23 ± 15.27
Lr15	−45.25 ± 4.41	−102.80 ± 7.57 *	−32.13 ± 9.21	−70.35 ± 12.04
Lr16	−54.40 ± 9.49	−74.60 ± 10.24	−34.53 ± 2.40	−43.28 ± 2.21
Lr17	−33.95 ± 2.67	−56.70 ± 2.28 *	−41.65 ± 6.02	−42.75 ± 2.34
Lr18	−105.00 ± 3.92	−89.60 ± 5.99	−102.23 ± 7.80	−158.25 ± 7.80 *#
Lr19	−138.25 ± 3.86	−96.98 ± 9.05 *	−106.23 ± 25.49	−116.00 ± 8.12
Lr20	−111.00 ± 3.56	−96.23 ± 13.50	−73.75 ± 17.44	−84.68 ± 6.49
Lr21	−111.50 ± 4.36	−66.10 ± 15.55	−115.50 ± 6.45	−96.78 ± 6.73
Lr22	−47.45 ± 1.71	−43.63 ± 6.46	−75.05 ± 1.66 #	−49.48 ± 10.10

Data are presented as mean ± SD (n = 4 independent formulation replicates). * indicates a significant difference from the corresponding value before UAP at the same storage time; # indicates a significant difference from the corresponding value at day 0 under the same UAP condition (paired Student’s *t*-test with Holm adjustment for multiple comparisons, adjusted *p* < 0.05).

## Data Availability

Data is contained within the article.

## Abbreviations

The following abbreviations are used in this manuscript:

UAP	Ultrasound-assisted processing
PDI	Polydispersity index
DLS	Dynamic light scattering
ELS	Electrophoretic light scattering
LC–MS	Liquid chromatography–mass spectrometry
ESI	Electrospray ionization
SD	Standard deviation
